# Structure–Activity Relationships between the State of Silver on Different Supports and Their I_2_ and CH_3_I Adsorption Properties

**DOI:** 10.3390/nano11051300

**Published:** 2021-05-14

**Authors:** Bruno Azambre, Mouheb Chebbi, Nagham Ibrahim

**Affiliations:** 1 Laboratoire de Chimie et Physique-Approche Multi-Echelle des Milieux Complexes (LCP-A2MC-EA n°4632), Institut Jean-Barriol FR2843 CNRS, ICPM, Université de Lorraine, 1, Bd Arago, F-57500 Saint-Avold, France; Nagham_ibrahim5@hotmail.com; 2 Institut de Radioprotection et de Sûreté Nucléaire (IRSN), PSN-RES, Saclay, 91192 Gif-sur-Yvette, France; mouheb.chebbi@irsn.fr

**Keywords:** iodine, methyl iodide, zeolite, ceria, alumina, silver iodide

## Abstract

In this study, the performances of silver-impregnated adsorbents prepared from different host supports (SBA-15, alumina, ceria, and faujasite Y zeolite) and calcined or not at 500 °C (1 h) were compared for the capture of I_2_ and CH_3_I. By keeping the silver content rather similar (about 15–17 wt %) among the sorbents, it was possible to assess the effect of silver dispersion and speciation on the adsorption capacities measured for both adsorbates. In a first part, several characterization techniques (XRD, DRS-UV-Vis, TEM, etc.) were used to probe the state of silver in the calcined and non-calcined materials. It was found that the characteristics of silver species are strongly influenced by the thermal treatment, the presence or absence of exchange sites, and the stability of the supports. Silver agglomeration was enhanced after calcination at 500 °C especially for supports bearing no exchange sites (SBA-15) or no ordered pores (alumina and ceria). Then, the adsorption performances of the studied silver sorbents were discussed in relation with their physicochemical characteristics. After-test characterizations were useful to assess the proportion of silver species that have reacted with CH_3_I and I_2_ to yield AgI precipitates. Depending on the adsorbate, different trends were obtained. I_2_ adsorption/reaction with silver sites was found to be quantitative (I/Ag ≈1), whatever the silver speciation and dispersion on the support. By contrast, a high proportion of cationic silver species was found essential to increase CH_3_I adsorption (I/Ag about 0.6–0.7 against 0.2–0.3 for Ag agglomerated species).

## 1. Introduction

Accidental releases of radiotoxic species from nuclear plants to the environment are known to cause serious damages. Among the most hazardous radionuclides, iodine is of specific interest due to its high radiological impact associated with its ability to exist both as aerosols and volatile species, namely I_2_ and CH_3_I [1,2,3]. Indeed, emissions of radioiodine could occur in the course of a severe nuclear accident or during the reprocessing of nuclear fuels. The remediation of contaminated waters also represents a serious concern in that respect [4]. One efficient technological approach to overcome such releases is to trap the released iodine forms onto a sorbent. Owing to the possibility of forming thermally stable and insoluble AgI precipitates, silver-loaded materials were identified as efficient candidates for the trapping of methyl iodide, molecular iodine, and iodide ions at an industrial scale [5,6,7,8]. The most studied silver-based adsorbents for the capture of volatile iodine are silver zeolites and silver supported on various forms of silica. A great R&D effort has been devoted to silver zeolites for over 50 years thanks to their thermal stability, radiation resistance, as well as tunable properties (structure, pore size and connectivity, Si/Al ratio, etc.) [9,10]. Indeed, we and others have tested a large panel of silver zeolite configurations (nature of cation, Si/Al ratio, zeolitic structure) by experimental and computational means in order to gain insights about the most prominent parameters affecting the retention of volatile iodine [11,12,13,14,15,16,17,18]. Some silver zeolites are now still currently investigated at industrial scale in France for their future incorporation in Filtered Containment Venting Systems (FCVS). In case of accident, such FCVS technology aims to prevent containment damage to nuclear power plants while affording simultaneously a reliable retention of radioactive products [19]. On the other hand, other kinds of silver adsorbents, such as silver nitrate deposited on silica and alumina, were also implemented on nuclear fuel reprocessing plants in Germany [20] and in Japan [21], respectively, for the capture of radioiodine. These adsorbents were reported to form trapping products such as silver iodide (AgI) or silver iodate (AgIO_3_), but without any experimental evidence for the latter [22]. Overall, these sorbents displayed moderate decontamination factors (about 10^2^) towards I_2_ and CH_3_I compared with silver zeolites (10^3^–10^4^) at 150 °C [2,10] but a lower cost (by a factor of 3 to 10) [23]. Silver-functionalized silica and aluminosilicate aerogels are also currently developed for iodine capture from the off-gas of a nuclear fuel reprocessing plant [24]. These materials are characterized by high specific surface area and exhibited interesting adsorption performances, with adsorption capacities for molecular iodine equal or superior to silver mordenite and faujasites. In addition to these industrializable adsorbents, a few studies [11,25] also exist on silver supported on mesoporous silicas (of SBA-15 and MCM-41 types) thanks to their high specific surface areas, high hydrothermal stability, as well as tunable pore size in the mesopore range (2–10 nm). Hence, a better accessibility to silver sites and a lesser sensitivity to pore blockage while forming AgI precipitates could be expected for these materials in comparison with microporous zeolites [26]. For a Ag/MCM-41 system, it was reported that silver introduction and its subsequent reduction by NaBH_4_ in the MCM-41 material enhanced adsorption performances towards I_2_ (T = 35 °C), with an (possible overestimated) adsorption capacity of 760–770 mg/g against about 100–130 mg/g without silver [25]. By contrast, we found that the impregnation of silver nitrate on SBA-15 support (up to 40 wt %) did not lead to a significant improvement in adsorption performances in comparison with silver-exchanged zeolites, especially for CH_3_I [11]. Although many studies are available in the open and industrial literature about the performances of silver-based sorbents for the capture of iodine, the lack of adequate characterization data represents a strong limitation towards the understanding of the parameters governing the adsorption of I_2_ or CH_3_I from a material viewpoint. Only recently, the relationships existing between the adsorption performances and the main physicochemical characteristics of the materials were identified for silver zeolites [14,15,16]. In that respect, we found that the silver oxidation state (Ag° or Ag^+^) as well as its content and dispersion in the pore system of the host support are the most important parameters governing the adsorption performances for I_2_ or CH_3_I [17]. The present study aims to expand these findings by designing a comparative study to investigate different classes of silver-based materials on a common basis. Since it was established that the silver content had a major role on the adsorption capacities of volatile iodine species, we chose to keep this parameter rather similar for all tested materials in order to give us an additional chance to unravel the effect of silver speciation and dispersion separately from the silver content. Hence, an incipient wetness impregnation method was used to control the amount of silver deposited on the different supports bearing (faujasite) or having no exchange sites (alumina, ceria, and SBA-15 silica). For each support, the silver dispersion and oxidation state was further tuned using an additional thermal treatment. In the first part, a systematic characterization of these silver-impregnated materials was performed by several techniques (XRD, DRS-UV-Vis, TEM, etc.) in order to better assess the effects of the support and thermal treatments on the characteristics of impregnated silver species. In a second part, particular attention was devoted to discuss the adsorption performances of these adsorbents towards I_2_ and CH_3_I in relation with characterization data. After-test characterizations were also carried out in order to give some insights about the reactivity of silver species and AgI formation.

## 2. Materials and Methods

### 2.1. Parent Substrates and Silver Impregnation

#### 2.1.1. Synthesis of Parent SBA-15

The parent SBA-15 was synthesized at a scale of 10 g by a cooperative self-assembly mechanism using a protocol adapted from Zhao et al. [26]. The molar ratio used for synthesis is 1.0 TEOS/0.017 P123/5.5 HCl/172 H_2_O, and additional details about the procedure were already reported during our previous study [27]. Once the Pluronic P123 template was removed by calcination at 550 °C (2 °C/min, dwell time 6 h), the resulting SBA-15 material had a specific surface area of 783 m^2^/g and displayed a narrow size distribution of mesopores *ca* 7.5 nm (from BJH method on desorption branch). 

#### 2.1.2. Other Substrates

A commercial faujasite Y zeolite (Si/Al = 2.5, *CBV 300*, S_BET_ = 925 m^2^/g) was provided by ZEOLYST Int., Conshohocken, PA, USA under ammonium form and is denoted as NH_4_/Y (1.2). Commercial ceria (CeO_2_, SOLVAY, La Rochelle, France, S_BET_ = 235 m^2^/g) and alumina (Al_2_O_3_, SIGMA-ALDRICH, St Louis, MI, USA, *06300*, S_BET_ = 155 m^2^/g) were also studied for comparison purposes. 

#### 2.1.3. Preparation of Silver-Impregnated Materials

The preparation methods reported below as well as sample storage were carried out in the dark in order to avoid any photo-reduction of silver species. 

Silver was incorporated in the support materials through the Incipient Wetness Impregnation (IWI) method. For each support, the preparations were adapted in order to reach a nearly similar silver content (about 15–18 wt %). The used protocols consisted in incorporating several aliquots (100 µL each) of silver nitrate solution (Sigma-Aldrich, St. Louis, MO, USA, purity >99.9%) until pore saturation was achieved. The pore volume accessible to the solution as well as the AgNO_3_ concentration used for each support are reported in ESI (Table S1). After silver introduction, the prepared materials were dried under vacuum at 80 °C overnight. The obtained adsorbents will be denoted thereafter as *xAg/support*, where x is the mass percentage in silver as deduced from elemental analysis (see Table 1). 

The resulting adsorbents were either used directly or submitted to a calcination treatment performed at 500 °C (duration of 1 h) under air in a muffle furnace with a heating rate of 5 °C·min^−1^. This treatment was carried out in order to tune the state and dispersion of silver species in order to assess the influence of these parameters on I_2_ and CH_3_I adsorption properties. Then, the obtained adsorbents are denoted in the following as *xAg/support-calc.*

### 2.2. Characterization Techniques

Analyses related to the silver content in the different sorbents were performed using an Atomic Absorption Spectrometer (Ice^tm^ 3300 THERMO FISHER SCIENTIC, Waltham, MA, USA), equipped with a AgCuCr lamp, working at 75% energy with a current of 10 mA. Before analysis, the silver contained in the materials was dissolved using a concentrated HNO_3_ solution (see Table S2 for details), the resulting slurries were filtrated and further diluted at the appropriate concentration for analysis (338 nm, Y = 0.07765X−0.0077). 

Powder X-ray diffraction (PXRD) measurements were carried out before and after iodine adsorption tests using a Miniflex II diffractometer from RIGAKU (Tokyo, Japan) with the CuKα radiation. PXRD patterns were recorded for 2θ values ranging from 5 to 70°, using increments of 0.02° and a counting time of 2 s. The mean size of Ag° or AgI particles detected by XRD, was deduced from the Debye-Scherrer law (see Figure S3 in Supplementary Information). 

DR UV-Vis spectra were collected on a *Cary 4000 UV-Vis* (AGILENT TECHNOLOGIES, Santa Clara, CA, USA) spectrometer equipped with a double monochromator and *DRA900* integrating sphere also supplied by Agilent. Spectra were registered between 200 and 800 nm, with a resolution of 2 nm and a scan rate of 600 nm·min^−1^. Reflectance spectra were plotted in pseudo-absorbance mode, after correction with a *Spectralon standard* (taken as reference).

Transmission Electron Microscopy (TEM) studies were performed at the service of microscopy hosted at Institut Jean Lamour (CNRS- Nancy) using a *CM200* microscope (PHILIPS, Amsterdam, The Netherlands) equipped with EDX detector. Samples were simply dispersed on a carbon film under dry conditions in order to avoid any reduction of silver species by a solvent.

### 2.3. Iodine Adsorption Tests

#### 2.3.1. I_2_ adsorption Tests

Adsorption tests with molecular iodine were performed using the different silver-impregnated sorbents in liquid medium. Cyclohexane was selected as a solvent owing to its ability to solubilize large amounts of iodine crystals (2.719 g I_2_ for 100 g of C_6_H_12_ at 25 °C). In addition, Cyclohexane–I_2_ solutions display in UV-visible range two absorption maxima at 223 and 523 nm, the latter being responsible of the pink-violet color. Adsorption tests were carried out at (20 ± 5 °C) using 75 mg of sorbent (m_ads_) contacted in dark with 50 mL of a 400 mg·L^−1^ solution prepared from I_2_ pellets (ALFA AESAR, Ward Hill, MA, USA, purity 99.5%). The slurry was stirred for a sufficiently long time (about 24–48 h) to ensure reaching the adsorption equilibrium. Then, the adsorbent particles loaded with iodine were separated by filtration, and the solid was recovered for further characterization. Initial and final I_2_ concentrations were measured both on the initial solution and after 24–48 h exposure to iodine using a double-beam *Cary 4000 UV-Vis* spectrophotometer (AGILENT TECHNOLOGIES, Santa Clara, CA, USA) with 1 cm quartz cuvettes thanks to the Beer’s law applied at 523 nm:

$$\left[I_{2}\right]=3.6244\times A_{523}.$$

Hence, the adsorption capacity at equilibrium was deduced from the following mass balance:

$$Q_{I2}=\frac{{\left[I_{2}\right]}_{0}-{\left[I_{2}\right]}_{f}}{m_{ads}}\times V_{solution}.$$

#### 2.3.2. CH_3_I Adsorption Tests

CH_3_I adsorption tests in liquid medium (cyclohexane) were also carried out according to a protocol rather similar to the one used for I_2_ tests. However, some precautions were undertaken in order to take into account the volatile character of CH_3_I (boiling point of about 43 °C): (i) the adsorption temperature was maintained to 10 ± 1 °C using a refrigerated bath; (ii) a blank experiment was performed systematically for each adsorption test in order to account for any loss of iodomethane by evaporation (Δ_blank_). Adsorption tests were conducted using an initial CH_3_I concentration of 450 mg·L^−1^. Colorless Cyclohexane-CH_3_I solutions were characterized by a unique absorption band at 258 nm. Here also, the solutions were carefully filtrated to recover the spent adsorbents for further characterization. Absorbance measurements allowed us to determine the initial CH_3_I concentrations and at equilibrium (48 h). The adsorption capacity at equilibrium was then deduced using the following formula:

$$Q_{CH3I}=\frac{A_{258\;\left(0\right)}-A_{258\;\left(f\right)}-\Delta_{blank}}{2.9064\times m_{ads}}\times V_{solution}.$$

## 3. Results and Discussion

### 3.1. Characterization of Silver-Impregnated Adsorbents 

#### 3.1.1. Silver Content

The main chemical and textural characteristic of the silver-containing sorbents vacuum-dried at 80 °C or calcined at 500 °C (1 h) are reported in Table 1. As expected, a rather similar silver content was measured by AAS for the different supports (values ranging from 14.4 to 18.6 wt %). 

Additional information about the location, electronic state, and morphology of silver species and silver nanoparticles will now be discussed from XRD, DRS-UV-Vis, and TEM analyses presented in the forthcoming sections. 

#### 3.1.2. Study of Silver Speciation and Dispersion before and after Calcination by X-Ray Diffraction (XRD) and DR-UV-Visible Spectroscopy (DRS-UV-Vis)

**Figure 1:**
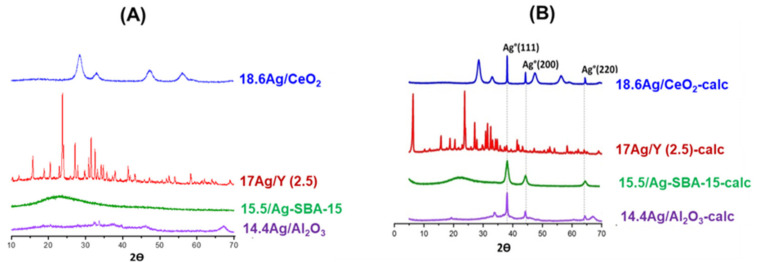
X-ray diffractograms of the silver-impregnated adsorbents: (**A**) samples dried at 80 °C, (**B**) samples calcined at 500 °C (1 h).

**XRD characterization.** The X-Ray diffractograms of the studied materials before and after calcination are presented in Figure 1. In the case of the materials having undergone only vacuum-drying at 80 °C (Figure 1A), the presence of silver crystalline phases (Ag_2_O or Ag°) cannot be established, indicating a rather good dispersion of the metal on the surface of the supports. For the 18.6 Ag/CeO_2_ sample, all of the four reflections are characteristic of the fluorite cubic structure of CeO_2_ (average crystallite size = 5.8 nm, as deduced from the Debye–Scherrer method). In fluorite structure, the cerium atoms occupy a face-centered cubic lattice whereas the oxygen atoms located in tetrahedral sites are known to have a high mobility [28]. The diffraction peaks observed for 17 Ag/Y (2.5) are typical of the faujasite structure (three-dimensional arrangement of AlO_4_^−^ and SiO_4_ tetrahedra forming 1.1 nm supercages separated by 0.74 nm windows). Owing to its low Si/Al ratio (2.5), this zeolite possesses a high Cation Exchange Capacity (CEC = 3.55 meq/g), which is defined as the amount of positive charge that can be exchanged per mass of zeolite [29]. Hence, most of the silver is expected to be rather finely dispersed within the internal porosity (mainly as Ag^+^ cations located at exchange sites). In the case of 14.4 Ag/Al_2_O_3_, the broad reflections hardly visible at 2*θ* = 37.02, 46.25 and 67.06° are ascribed to the *(311)*, *(400)*, and *(440)* reflections of a poorly crystallized γ-alumina phase. Nevertheless, some uncertainties remained, since the main peaks of metallic silver could partly overlap with those of the support [30,31]. Other small peaks cannot be assigned to a silver or alumina related phase and probably correspond to some impurities in the commercial alumina support.

After calcination at 500 °C (1 h, Figure 1B), the diffractograms of the different adsorbents display new reflections, except for Ag/Y zeolite. The peaks located at {2θ = 38.1^°^; *(111)*}, {2θ = 44.3°; *(200)*}, and {64.4°; *(220)*} are characteristic of the presence of Ag^°^ metal nanoparticles [32]. The formation of these metallic species during the thermal treatment is responsible for their brownish (15.5 Ag/SBA-15-calc), grey (18.6 Ag/CeO_2_-calc), or black (14.4 Ag/Al_2_O_3_-calc) colors after calcination. Hence, among the studied adsorbents, only the diffractogram related to Ag-zeolite treated at 500 °C (17 Ag/Y-calc) shows the absence of any detectable silver nanoparticles. Our previous study [17] had also confirmed the ability of this low Si/Al ratio Y zeolite to stabilize and disperse silver species within its microporous framework, and this, whatever the preparation method (ion exchange vs. impregnation). For the other investigated materials, the lack of exchange sites leads to a higher surface mobility of the introduced silver species. Hence, the subsequent drying and thermal treatment at 500 °C induced the agglomeration as well as the self-reduction of these species [30]. 

In order to gain further insight on the effect of the support on these processes, the average size of silver nano-crystallites was estimated for each (calcined) adsorbent from the Debye–Scherrer method. Values are reported in Table 1. Except for the Y zeolite, the SBA-15 support is the one that seems to constrain the most the growth of silver nanoparticles. Due to the similarities between the average size of nano-crystallites (9 nm) and those of mesopores (7.5 nm), it seems that the coalescence and growth of silver nanoparticles is somewhat controlled by their confinement inside the mesoporosity. In that respect, the materials bearing no internal pores, i.e., ceria and alumina, behave differently. Agglomerated silver forms were found to be more important for alumina (average size 27 nm) and especially promoted for cerium oxide (106 nm). For the latter, it is possible that the existence of large silver nanoparticles is favored by the mild sintering of the support at 500 °C. Indeed, it was found that the average crystallite size of ceria nanoparticles increased from 5.8 to 8.2 nm after calcination at 500 °C.

Overall, the apparent dispersion of silver species from XRD after calcination follows the order: 17 Ag/Y-calc > 15.5 Ag/SBA-15-calc > 14.4 Ag/Al_2_O_3_-calc > 18.6 Ag/CeO_2_-calc. 

**DRS-UV-Vis.** DR-UV-Vis spectroscopy was used in order to provide complementary information on the electronic state of silver species incorporated within the investigated supports prior to calcination. According to the literature studies by us and others [14,15,16,17,30,33,34,35], the absorptions relevant to Ag^+^ cations in different environments are usually found in the range 200–240 nm, whereas the plasmon resonance of silver nanoparticles is responsible of broad absorption bands in the visible region. Other signals are more difficult to assign, but it is generally admitted that charged and metallic clusters (Ag_n_^δ+^ and Ag^°^_m_) yield absorptions at 240–280 nm and 280–350 nm, respectively [14,15,16,17,30,33,34,35].

**Figure 2:**
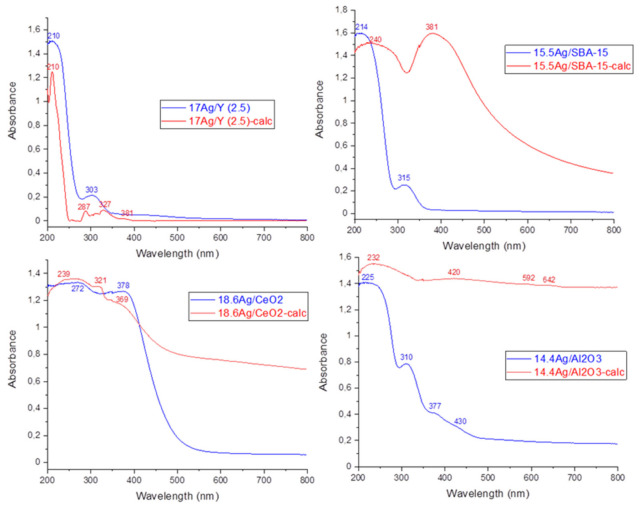
DRS-UV-Vis spectra of silver impregnated supports: only vacuum-dried at 80 °C (blue); calcined at 500 °C (1 h) (red).

DRS-UV-Vis spectra of the materials vacuum-dried at 80 °C and calcined at 500 °C (1 h) are compared on Figure 2. Starting by the 17 Ag/Y zeolite and 15.5 Ag/SBA-15 mesoporous silica, the main absorptions observed after drying at 80 °C are located at 210 and 214 nm, respectively (blue curves on Figure 2). These signals are assigned to Ag^+^ cations in the cavities and surrounded by water molecules. Moreover, the secondary maxima at 303–315 nm arise from small silver clusters of a few atoms and located in the cavities (designated as Ag^°^_m_ because they carry a weak charge, close to metallic silver). For these two materials, the calcination treatment at 500 °C (red spectra on Figure 2) induced distinct effects in function of the presence (Y zeolite with Si/Al ratio = 2.5) or absence (SBA-15) of exchange sites. For the 17 Ag/Y-calc sample, the absorptions are rather close to those observed for the non-calcined zeolite. However, the band assigned to Ag^+^ cations at 210 nm looks now sharper due to the elimination of bonded H_2_O molecules. For 15.5 Ag/SBA-15-calc, important changes could be noticed. The band previously assigned to Ag^+^ cations in hydrated/nitrate environment is now broadened and red-shifted. This witnesses of the existence of partially reduced silver species of a few atoms, such as Ag_n_^δ+^ and Ag^°^_m._ Moreover, the main signal is now related to the plasmon resonance of silver nanoparticles at 381 nm and spreading in the visible region. This strong absorption is responsible of the brownish color of the calcined sample and clearly witnesses of the existence of strong auto-reduction phenomena during calcination. For the 17 Ag/Y-calc zeolite which remained colorless after calcination, this reduction does not occur to a great extent, because the silver species in oxidized form are somewhat stabilized by the presence of exchange sites and protons in the faujasite structure. Nevertheless, weak signals are also visible at 287, 327, and 381 nm due to the partial auto-reduction of Ag^+^ cations promoted by the dehydration process [36]. These silver species (Ag^+^, Ag_n_^δ+^, and Ag^°^_m_) are expected to be confined within the internal microporosity of the 17 Ag/Y-calc zeolite.

Coming now to the silver-impregnated alumina sample before calcination (blue spectrum, 14.4 Ag/Al_2_O_3_), both the presence of a broad adsorption at 225 nm and other maxima at 310, 377, and 430 nm are clearly visible. The former is attributed to silver nitrate species (silver nitrate in solution has a signal at 226 nm), whereas the others belong to other forms of partially reduced silver. By comparison with Ag/SBA-15, it seems that reduction processes are more promoted for Ag/Al_2_O_3_. This trend is even more clearly observed after calcination. Owing to the black color of the 14.4 Ag/Al_2_O_3_-calc sample, an almost total absorption occurred in the visible region. 

As an *n*-type semi-conductor, the CeO_2_ support exhibits an absorption edge in the visible region, which is also responsible for its pale yellow color. This feature dominates the DR-UV-Vis spectra of the 18.6 Ag/CeO_2_ and 18.6 Ag/CeO_2_-calc samples and is attributed to a charge-transfer transition of the oxygen ions to the cerium ions (O_2_^−^ → Ce^4+^) [28]). Due to the strong absorption of the support below 378 nm, it is somewhat difficult to accurately analyze the absorptions of impregnated silver species (blue spectrum on Figure 2). Nevertheless, it seems reasonable that most of silver species are present as Ag^+^ cations (signals overlapped with those of Ce^4+^/Ce^3+^ cations), small clusters, or very small nanoparticles (absorption at 378 nm), in a way rather similar to silver alumina. Following calcination at 500 °C (red curve), a rather deep reduction and sintering of silver species is observed, as shown by the development of novel absorptions covering the whole visible range. 

To sum up, it seems that silver speciation and dispersion on the different supports are rather strongly dependent on the thermal conditions used during preparation, the presence/absence of exchange sites, the reducible character, and the stability of the support material. After drying at 80 °C (blue spectra), most of the silver is present as Ag^+^ cations and to a minor extent as partially reduced silver species, a highest proportion of reduced silver species being observed on alumina and ceria supports due to the absence of exchange sites and the absence of internal porosity. After calcination at 500 °C (1 h), agglomeration of silver as nanoparticles with metallic character is observed clearly for SBA-15, ceria, and alumina. By contrast, Ag^+^ cations and more dispersed forms of silver are still the dominant species for the Y zeolite thanks to its high CEC. These observations seem to be in agreement with porosimetric analysis of these silver-containing materials before and after calcination. Indeed, a general decrease of textural properties is observed for all silver-loaded sorbents (calcined or not) in comparison with their parent materials (Figure S1). Moreover, this process was found to be more important for supports bearing no exchange sites or no ordered pores, because in that case, the silver mobility is increased, leading to accelerated coalescence and sintering during drying/calcination [30].

**Figure 3:**
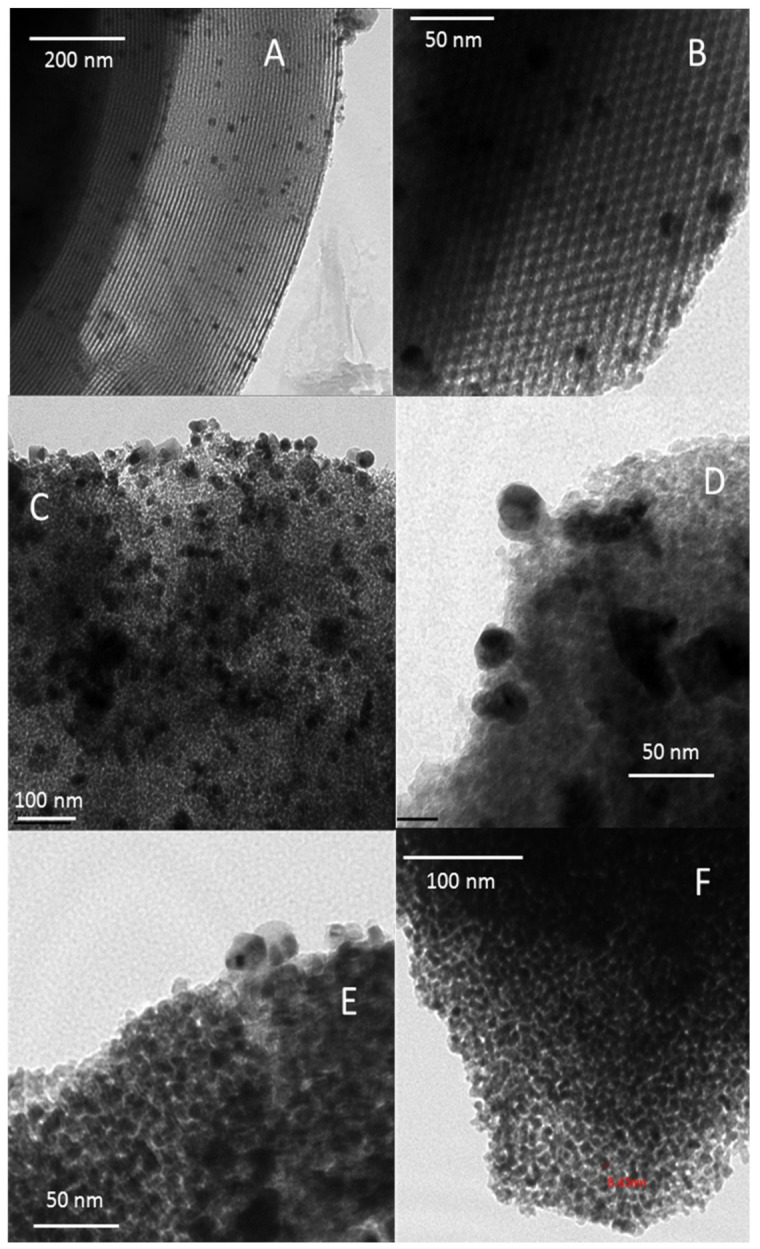
TEM micrographs of the calcined samples: 15.5 Ag/SBA-15-calc (**A**,**B**), 14.4 Ag/Al_2_O_3_-calc (**C**,**D**), 18.6 Ag/CeO_2_-calc (**E**,**F**).

**Transmission Electron Microscopy (TEM).** Characterization by TEM was also achieved in order to assess the size distribution and morphological features of silver nanoparticles existing on the different host supports. A selection of micrographs is presented in Figure 3 for calcined adsorbents bearing those nanoparticles, i.e., silver-impregnated mesoporous silica, cerium oxide and alumina, respectively. It is important to remember that non-calcined samples could not be studied by this technique, because of the rapid sintering of dispersed forms of silver to nanoparticles due to electron beam and ultra-vacuum conditions [14,33].

The organized mesoporous character of Ag-impregnated SBA-15 can be directly assessed by TEM (Figure 3A,B) in agreement with the porosimetric analysis (type IV isotherm with H1 hysteresis, Figure S1). More particularly, different views were obtained depending on the orientation of the electron beam compared to the pores axis. Indeed, the relatively ordered array of mesopores (diameter 7–8 nm) is observed when they are directed perpendicularly to the electron beam (Figure 3A). By contrast, the hexagonal honeycomb structure of the channels can be evidenced when the main axis of the pores is parallel to the electron beam (Figure 3B). The silicic walls of the pores appear in dark gray on the images (Figure 3A,B), while the voids formed by the elimination of organic surfactants appear clear (lower electron density). Moreover, it appears that most of silver nanoparticles formed in the course of calcination process remained located in the mesoporous channels. However, the coalescence of silver species has sometimes induced some enlargement and damages to the mesopores. Moreover, the distribution in size of these nanoparticles (5–12 nm in average, Figure 3A) matches rather well with the average crystallite size computed by the Debye–Scherrer method using XRD (9.1 nm). Overall, this confirms that the particle growth was constrained by free space in mesopores. 

For the silver-impregnated alumina and ceria materials (Figure 3C–F), the silver nanoparticles are bigger and less homogeneously distributed on the support surface. This is not surprising considering the absence of internal porosity and lower specific surface area for ceria and alumina supports in comparison with SBA-15 silica and Y zeolite. Hence, such characteristics are expected to promote the agglomeration process of silver. Particularly, the γ-alumina support (Figure 3D) is constituted by crystallites having an irregular morphology and a size of a few nm. In the 14.4 Ag/Al_2_O_3_-calc sample (Figure 3C,D), the silver nanoparticles correspond to the black areas in the images. Most of them have a size of 20–30 nm but both smaller entities and larger silver particles are present. Surprisingly, the presence of cubic copper crystallites were also observed at some many places within the sample. The 18.6 Ag/CeO_2_-calc sample (Figure 3E,F) is constituted from small interconnected CeO_2_ crystallites of about 5–10 nm, which agrees with XRD data (8.2 nm in average after calcination). As already reported in previous studies, the mesoporosity of this sample arises from the vacant spaces between nanocrystallites. This fact may be also witnessed from the presence of hysteresis in the region of P/P_0_ 0.5–0.8 in the associated N_2_ adsorption/desorption isotherm (Figure S1). The black areas observed at different locations in the image correspond to silver nanoparticles having a wide size distribution, but the poor contrast with the ceria support makes it difficult to visualize them accurately. 

In the next sections, the performances of these sorbents for the adsorption of I_2_ and CH_3_I will be presented. More particularly, some relationships will be established between the silver speciation and the uptake of iodine species.

### 3.2. Adsorption Properties for Molecular Iodine and Iodomethane

#### Comparison of Adsorption Capacities

Before addressing the adsorption of volatile iodine species on the different silver-containing adsorbents, it has to be mentioned that CH_3_I and I_2_ adsorption on the supports alone was in most cases negligible, i.e., in the range 0–10 mg/g. Nevertheless, slightly higher adsorption capacities were noticed in some preliminary experiments. Residual adsorption is likely to be explained by some interactions existing between iodine species and organics coming from surface adsorption or left after synthesis and not by direct interactions with the sites of the supports.

**Figure 4:**
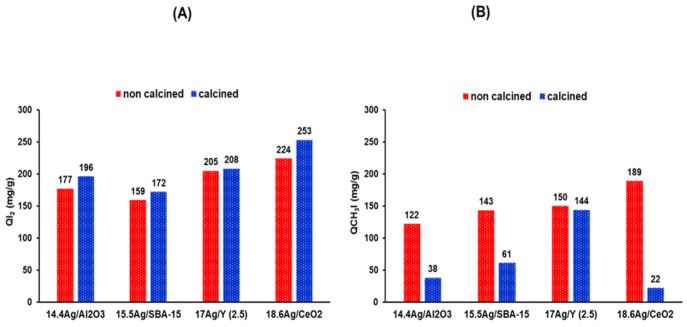
Influence of the thermal treatment on the performances of the investigated silver-impregnated adsorbents towards the capture of I_2_ (**A**) and CH_3_I (**B**).

**I_2_ adsorption**. Adsorption capacities for molecular iodine were measured from batch sorption tests performed at the following conditions: T = (20 ± 5) °C, m_ads_ = 75 mg, [I_2_]_0_ = 400 mg·L^−1^, and V_solution_ = 50 mL, 24 h stirring in dark. The amounts of adsorbed I_2_ measured for the different silver-impregnated supports (before and after calcination) are gathered in Figure 4A as well as in Table 2. 

Depending on the type of adsorbent, the adsorption capacities for molecular iodine lay in the range 159–253 mg/g. Nevertheless, for the same class of adsorbent (ceria, alumina, zeolite faujasite Y, or silica SBA-15), the adsorbed amounts in I_2_ before and after calcination were found to be generally close, only a slight increase being noticed after calcination. 

Comparing now the adsorption capacities with the physicochemical properties of each investigated material, it can be deduced that the oxidation state of silver and its dispersion on the support have a negligible impact on the adsorption of molecular iodine. Indeed, it was shown in the characterization section that silver entities in all investigated materials exist mainly in oxidized and dispersed state prior to calcination (with charged or metallic clusters of small size). However, and except for 17 Ag/Y-calc zeolite, a major part of these entities convert to reduced metal nanoparticles after calcination at 500 °C (1 h). Considering the large silver nanoparticles found on alumina and ceria after calcination (26 and 106 nm from XRD, respectively), it seems that mass-transfer limitations from the surface to the bulk of silver nanoparticles are not an obstacle to the adsorption of molecular iodine. In other words, whatever the silver speciation and dispersion on the support, the iodine uptake process is quantitative. This can be clearly evidenced considering the molar I/Ag ratio. In general, the data reported in the Table 2 indicate an I/Ag ratio close to unity for almost all sorbent materials (both calcined and not calcined). Hence, nearly all silver atoms were used by iodine to form AgI precipitates. These data confirm that the affinity of I_2_ towards silver is so strong that the thermodynamic driving force of iodine adsorption is almost not affected by the silver initial state neither by the support nature. 

Our data seem to agree well with some literature studies. In a comprehensive study by Funke et al. [37], the parameters that could potentially influence the kinetics of I_2_/Ag reaction for silver educts in aqueous solutions were systematically studied. Two different regimes were identified using a first-order kinetic model (an initial rapid uptake of iodine by silver to form AgI during the 5 first minutes and then a slower process (by a factor of 3) due to transport limitations. No dependencies of the rate constants were found on the parameters temperature (varied between 25 and 160 °C), initial I_2_ concentration (in ratio 10^−2^ to 10^−4^ in respect with silver), type of silver material (powders with different surface areas, plates, coarse shreddings of control rod material), and pretreatment of the silver educt (hydrogen reduction) prior to the tests. However, the stirring of the reaction solution generally enhanced the kinetics, highlighting the importance of mass transfer. In a DFT surface science study, Andryushechkin et al. [38] have studied I_2_ adsorption on a model Ag *(100)* surface at 300 K. It was shown that the dissociation of iodine proceeds without activation barrier with I atoms initially sitting on fourfold hollow sites. After saturation of the chemisorbed layer, the nucleation and growth of the flat silver iodide islands start near the step edges and consisted of four hexagonal atomic planes parallel to the substrate surface. They are arranged in a sandwich-like structure, containing two coupled silver planes in the middle and iodine planes on each side. Then, the process continues with the formation of AgI 3D epitaxial layers, this being very facilitated by the high mobility of iodine on transition metal surfaces [37].

Moreover, the present results appear also to be consistent with our previous work dedicated to the sorption performances of Ag-zeolites towards I_2_ [16]. Indeed, it was shown that the adsorption of molecular iodine can proceed both on Ag^+^ cations (at the exchange sites) in the porosity or on metal nanoparticles mainly located at the external surface. In both cases, AgI precipitates were also found to be formed. Moreover, the determination of quasi-linear correlations between the elemental silver content in various zeolitic structures (FAU X and Y, MOR, *BEA, FER, and MFI) and I_2_ adsorption capacities demonstrates that this parameter is much more influencing than silver speciation or the porous characteristics of the material [16]. By comparing now the silver contents of the different classes of adsorbents, it appears that the amounts of adsorbed I_2_ here also globally increase with the amount of silver. However, it can be noticed that alumina with 14.4 wt % silver displays a higher I_2_ adsorption capacity than mesoporous silica with 15.5 wt % silver. This may be explained by the presence of copper impurities in the commercial alumina support, which were also evidenced by TEM-EDX (not shown here). Indeed, copper species can act as supplementary adsorption sites for I_2_, as also shown in our previous study devoted to the influence of zeolitic cation on iodine retention [17]. 

**Figure 5:**
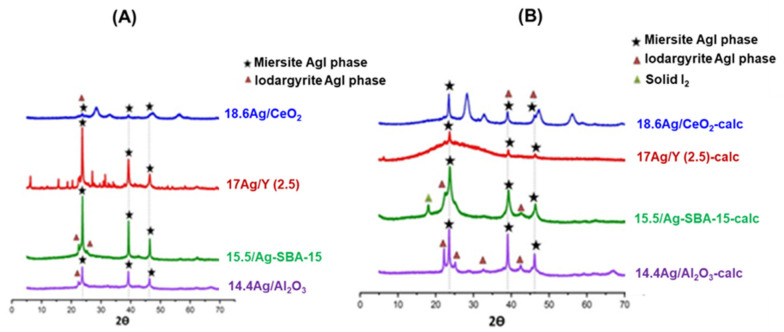
X-ray diffractograms of silver-impregnated adsorbents after I_2_ adsorption test: (**A**) samples dried at 80 °C, (**B**) samples calcined at 500 °C (1 h).

A closer look at the data shown on Figure 5A indicates that calcined materials display in general slightly higher adsorption capacities than the non-calcined ones. Although this remains speculative, it is possible that the water content in the samples before test (a parameter that cannot be easily controlled) may be slightly higher for the samples that have not undergone calcination, resulting in somewhat improved adsorption capacities compared for freshly calcined adsorbents (due to a slightly higher Ag content). 

To sum up, it can be concluded that silver speciation and its dispersion and accessibility on the different supports do not significantly impact the amount of adsorbed I_2_, and these results confirm that the most important factor in the case of molecular iodine remains the amount of silver incorporated (whatever its oxidation state and dispersion). These adsorption properties measured for molecular iodine will now be compared with measurement performed in the presence of CH_3_I.

**CH_3_I adsorption.** CH_3_I adsorption capacities were determined from batch sorption tests in cyclohexane at the following conditions: T = 10 °C, m_ads_ = 75 mg, [CH_3_I]_0_ = 450 mg·L^−1^ and V_solution_ = 50 mL, 24 h stirring in dark. The quantities of adsorbed CH_3_I measured for the different calcined and non-calcined supports are presented in Figure 4B and in Table 3. The observed trends for CH_3_I adsorption are as follows: (i) depending on the sample nature, adsorption capacities were affected to a much greater extent than for I_2_, with values ranging from 22 to 189 mg/g, indicating a greater sensitivity of CH_3_I adsorption to the nature of adsorption sites present in the different sorbents; (ii) A great gap, except for Ag/Y zeolite, is now observed between the non-calcined samples (dried at 80 °C) and the same materials calcined at 500 °C. The calcined samples have adsorption performances that are often much lower than their non-calcined counterparts (difference: ceria >> alumina > silica >>> zeolite); this decrease of adsorption performances is much more obvious for materials initially displaying the largest sizes of Ag^°^ nanoparticles (see characterization section). As a consequence, a fairly better use of silver can be inferred for the non-calcined samples (I/Ag atomic ratio about 0.7); however, the values are still slightly lower than those observed for I_2_ (I/Ag ratio close to 1). More striking, this ratio falls in the range 0.09–0.3 for calcined samples (except for the zeolite). 

Overall, silver cations, and possibly also other forms of silver entities within the internal porosity, appear to be more efficient than silver nanoparticles to ensure an efficient capture of CH_3_I. However and as stated before, this does not apply for I_2_ adsorption, since this molecule was adsorbed quantitatively whatever the speciation and dispersion of silver or the nature of the support. These trends are globally consistent with our previous findings and the literature [16,39]. In a previous study [17], we found that a silver Y zeolite with a high silver content (23 wt %) and Si/Al ratio (40) was not efficient for CH_3_I trapping but a similar zeolite with similar Ag content but a much lower Si/Al ratio (2.5) was. Actually, the quasi-absence of exchange sites for the almost pure silica zeolite (with Si/Al = 40) made impossible to ensure a good dispersion of silver species, leading to poor CH_3_I adsorption capacity (23 mg/g). By contrast, silver nanoparticles were not formed at lower Si/Al ratio (2.5) due to the possibility to disperse silver as Ag^+^ cations in exchange positions, leading to much greater CH_3_I trapping under similar conditions (up to 210 mg/g) [17]. 

In the study of Funke et al. [37], the parameters that could potentially impact the kinetics of I^−^ adsorption by silver educts (from aqueous solution) were also examined. The type of Ag educt (powders, plate, etc.), the temperature, the initial pH, and I^−^ concentration were not found to influence the rate constant. However, the absence of stirring and more importantly, the inertization of the reaction suspension decrease the reaction rate or even prevent the reaction leading to AgI. This led the authors to conclude that from the chemical point of view, the I^−^/Ag reaction needs an oxidizing step. This statement, even if obtained from rather different experimental conditions (non-supported silver, I^−^ in aqueous solution instead of CH_3_I/cyclohexane) appears to be consistent with our findings. In our case, no H_2_ pretreatment was performed on the adsorbents prior to the tests or inertization during the tests. Hence, it seems likely that some oxidized silver species (in the form of Ag^+^/Ag_2_O layer at the surface of silver nanoparticles or at specific locations in the surface) remained at the surface of ceria, alumina, and SBA-15, leading to some CH_3_I capture. Nevertheless, other studies on model silver surfaces have also pointed out additional information for the adsorption and desorption of methyl iodide. In the case of metallic silver surface, it appears that the CH_3_I adsorption process is not much sensitive to the structure (the silver crystallographic plane). At 130 K on Ag *(111)*, it was found that below the monolayer coverage, CH_3_I molecules adsorbed via the halogen atom on silver sites. Then, a fraction of these molecules undergo dissociation, and the CH_3_ fragments combine to ethane, which desorbs below room temperature. Remaining I fragments remained adsorbed, forming eventually AgI 2D islands on the surface that are stable up to 800–900 K. Increasing CH_3_I adsorption above monolayer coverage resulted simply in very weakly bonded CH_3_I multilayers and did not increase AgI formation [40]. 

It is interesting here to observe that CH_3_I adsorption capacities decrease in relation to Ag° nanoparticles size estimated from XRD data (Table 1). In the following part, a particular attention will be devoted to the after-test characterization of silver-impregnated adsorbents exposed to molecular iodine or iodomethane. Analyses by XRD, DRS-UV-Vis, and TEM were conducted with the aim to gain insights on the role of silver state on retention mechanisms.

### 3.3. Characterizations Performed on the Spent Adsorbents after I_2_ and CH_3_I Tests

After being exposed to I_2_ in the previously described adsorption experiments, namely all adsorbents became yellow or pale-yellow (Table 2). This color is typical of silver iodide precipitates whose critical size is above a certain threshold i.e., in the nanometric to micrometric range. By contrast, smaller AgI entities (those confined in the porosity) remain colorless, since their band gap occurred in the ultraviolet region. The yellowish colour was also observed after CH_3_I tests, but mostly for the non-calcined samples (and the 17.7 Ag/Y-calc). Characterization of the spent adsorbents by various analytical techniques was carried out in order to gain further insights on the phenomena involved in I_2_ and CH_3_I retention by the different impregnated materials.

#### 3.3.1. X-Ray Diffraction Analyses of AgI Precipitates

**Figure 6:**
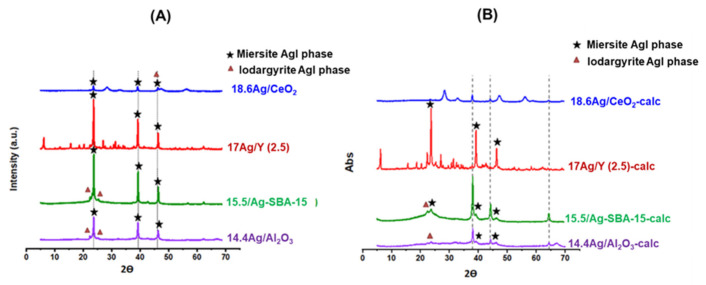
X-ray diffractograms of silver-impregnated adsorbents after CH_3_I adsorption tests: (**A**) before calcination and (**B**) after calcination. On Figure (**B**), dotted lines indicate the positions of the main reflections associated with remaining silver nanoparticles having not reacted with CH_3_I.

The diffractograms of the spent silver-containing adsorbents (SBA-15 silica, ceria, alumina, and faujasite zeolite) after I_2_ adsorption tests are presented in Figure 5A before and Figure 5B after calcination at 500 °C (1 h). Figure 6A,B are related to CH_3_I adsorption tests. In general, new reflections can be observed after test in comparison with the initial adsorbents. They are assigned to a mixture of silver iodide phases. The dominant AgI phase was found to be miersite (γ-AgI with cubic system and zinc-blende structure) and is characterized by three main diffraction peaks at 2θ = 23.72 *(111)*, 39.23 *(220)*, and 46.36° *(311)* (JCPDS No. 78-0641). In addition, the iodargyrite phase (β-AgI with hexagonal wurtzite structure, seven main peaks at 2θ = 22.39 *(100)*, 23.68 *(002)*, 25.37 *(101)*, 32.82 *(102)*, 39.30 *(110)*, 42.68 *(103)* and 46.40° *(112)* (JCPDS No. 78-1613)) was detected for most of the samples, but the diffraction peaks are much less intense than for the miersite phase, indicating that the latter predominates. 

In the case of I_2_ adsorption, AgI precipitates were systematically observed by XRD, whatever the adsorbent and the absence/presence of thermal treatment (calcination at 500 °C (1 h)). The facile detection of AgI precipitates for all samples exposed to iodine agrees well with the fact that relatively similar I_2_ adsorption capacities were obtained for the calcined and non-calcined adsorbents (Table 2 and Figure 4). Nevertheless, the reflections associated with the minor iodargyrite phase (hexagonal AgI) were more easily visible after calcination (especially for 14.4 Ag/Al_2_O_3_-calc and 15.5 Ag/SBA-15-calc) but they remained weaker compared with those of the miersite phase (cubic AgI). This indicates that the nature of silver entities initially present on the different supports, e.g., the type of reticular plane exposed for Ag nanoparticles may play a role on the nature of AgI precipitates formed following I_2_ adsorption, but only to a small extent. Moreover, it is likely that other kinds of AgI species, too small to be detected by XRD, may be still present in the pores of the adsorbents. In that respect, the co-existence of α-AgI in the micropores and γ-AgI on the external surface of an H_2_-prereduced Ag-MOR zeolite was evidenced for silver mordenite after adsorption of molecular iodine [41]. In the present study, the α-AgI phase could not be detected by XRD. This is possibly due to the limits of this technique for detecting intra-pore crystallites of small size. 

In addition to AgI precipitates, an additional peak at 18.0° (2θ) was detected in the XRD pattern of the 15.5 Ag/SBA-15-calc sample (Figure 5B). Without certainty, but considering the existence of ordered channels in mesoporous silica, this peak could possibly be in relation with the existence of iodine superstuctures confined in the channels of SBA-15.

On the other hand, it is interesting to note that the situation is different in the case of CH_3_I adsorption. With the exception of Ag/Y zeolite, AgI precipitates were barely detected for the calcined Ag/ceria, Ag/alumina, and Ag/SBA-15 samples. Instead, the peaks related to the presence of unreacted metallic silver nanoparticles are still well observed. By contrast, AgI-related phases (namely miersite) are clearly visible in the XRD patterns of the non-calcined adsorbents. Overall, these results agree well with those of quantitative adsorption tests (Figure 4), where it was shown that calcined Ag/ceria, Ag/alumina, and Ag/SBA-15 are not effective sorbents for CH_3_I and are rather effective for molecular iodine. In respect with characterization results, this clearly demonstrates that Ag° nanoparticles are far to be as efficient as Ag^+^ cations (and possibly small clusters) in trapping methyl iodide as AgI. This is also in agreement with the low I/Ag atomic ratio determined after CH_3_I tests for the calcined sample.

When it was possible, the mean size of AgI crystallites corresponding to the major miersite phase was calculated from the Debye–Scherrer method and is reported in Table 2. In general, the average AgI crystallite size exceeds the pore size of the materials and also the initial size of silver entities in the non-calcined and calcined materials. This is due to coalescence phenomena involving primary AgI or (AgI)_n_ entities, which take place both in and out of the pores. Despite precautions were taken, it is possible that external factors such as light and humid air play a role on coalescence phenomena. Nevertheless, for the faujasite Ag/Y zeolite, the calcination treatment at 500 °C (1 h) had apparently no impact on the AgI polymorph observed (γ or β) neither on the mean size of γ-AgI crystallites (37 vs. 35 nm before/after calcination). Hence, this seems in agreement with characterization results, which have shown a rather similar silver speciation before and after calcination. By contrast, a significant effect of calcination can be assessed in the case of Ag/SBA-15. For the calcined sample, the mean size of AgI crystallites (D_AgI_ = 16 nm, Table 2) does not exceed much the size of Ag° nanoparticles (9 nm), However, for the non-calcined sorbent bearing initially dispersed Ag^+^ cations, these AgI crystallites are much larger (D_AgI_ = 64 nm). Hence, there is no relationship between the initial size of silver entities and those of AgI crystallites, especially when the former are in a dispersed state. Rather, the higher intrinsic mobility of Ag^+^ cations and other dispersed silver entities (in comparison with the mobility of silver nanoparticles) may be a parameter that promotes the accelerated coalescence and growth of large AgI domains outside of the pores. In other words, it seems that metal nanoparticles react progressively with iodine through a solid-state diffusion process to give AgI particles of roughly comparable size with limited agglomeration, whereas the presence of dispersed and highly mobile silver within the channels seems to favor coalescence processes. 

#### 3.3.2. DRS-UV-Vis and TEM Analyses of AgI Precipitates 

**DRS-UV-Vis**. When enough samples could be collected after I_2_ and CH_3_I adsorption tests*,* additional DRS-UV-Vis characterizations were performed in order to give complementary information to XRD on the nature of AgI entities. 

**Figure 7:**
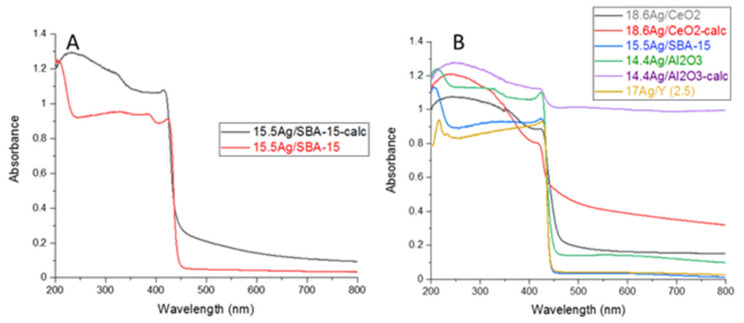
DR-UV-Vis spectra of some selected sorbents after I_2_ (**A**) and CH_3_I (**B**) adsorption tests.

The reference crystalline commercial micrometric AgI (Alfa Aesar, *114119*, not shown here but visible in [12,14]) has an absorption edge at 432 nm in agreement with its yellowish color. As an n-type semi-conductor, it has a band gap at 2.75 eV [12,42]. The DR-UV-Vis spectra 15.5 Ag/SBA-15 and 15.5 Ag/SBA-15-calc materials obtained after I_2_ adsorption also exhibit a characteristic absorption edge, which is slightly blue-shifted in comparison with the reference, at 421 and 414 nm, respectively (Figure 7A). This blueshift was associated in our previous studies to the existence of AgI crystalline precipitates of smaller size in comparison with the reference. For instance, nanometric or sub-nanometric AgI entities located in the pores are expected to display an absorption edge below 400 nm due to quantum confinement effects and are therefore colorless. Hence, the less pronounced blueshift of the absorption edge (421 nm) for the non-calcined Ag/SBA-15 could be paralleled with XRD characterizations, indicating the enhancement of AgI sintering phenomena for precipitates formed from well-dispersed and mobile silver cations instead of silver nanoparticles. Moreover, the DRS-UV-Vis technique seems useful in probing the presence of very small AgI entities not detected by XRD. Hence, the spectra of the tested adsorbents (both calcined and non-calcined) also indicated absorption bands located 285 and 345 nm, respectively, which could be attributed to very small AgI (quasi-molecular) entities (Figure 7A).

On the other hand, and by comparing the spectrum of the non-calcined 15.5 Ag/SBA-15 sample (Figure 7A), red) with the initial material not exposed to iodine (Figure 2, blue), it seems that some Ag^+^ cations remained unaltered. For the calcined material (Figure 7A), black), the presence of a broad absorption at wavelengths above the absorption edge witnesses of the presence of residual silver nanoparticles; however, it is in lower concentration compared with the unexposed material (Figure 2, red). Overall, these data could be linked to adsorption results, which have shown that the molar I/Ag ratio after I_2_ tests remained below unity (0.86 and 0.94, respectively) for Ag/SBA-15 samples. Hence, it seems that some silver sites, probably deeply buried in the pores, could not be accessible to iodine and react to form AgI precipitates. 

**Figure 8:**
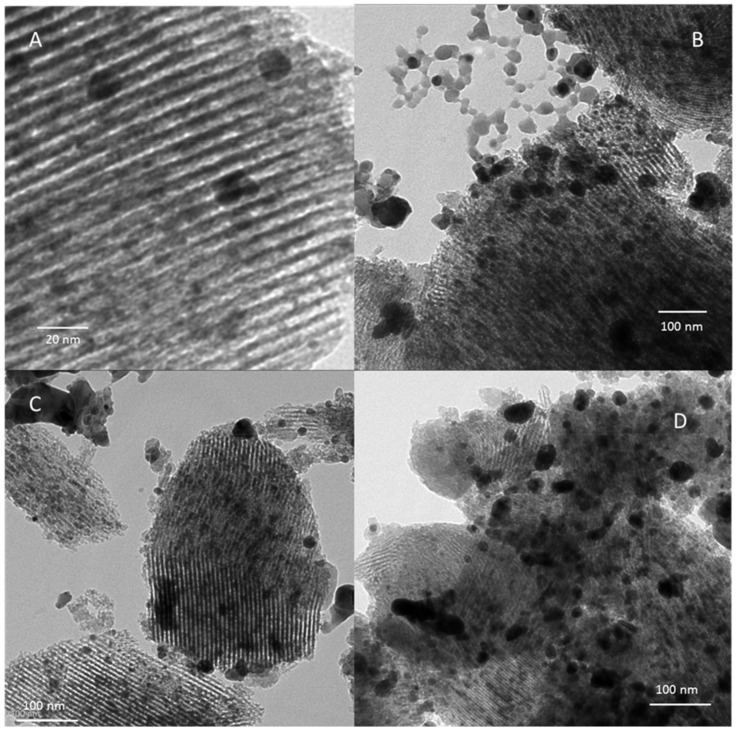
TEM micrographs of the calcined 15.5 Ag/SBA-15-calc material after I_2_ adsorption test. Images (**A**–**D**) represent different views of SBA-15 channels and the presence of AgI crystalline phases inside or outside of the pores.

The same samples (*15.5 Ag/SBA-15, 15.5 Ag/SBA-15-calc*) were also subjected to analysis by electron microscopy (TEM). The non-calcined adsorbent was found to be rather unstable under the electron beam and vacuum conditions in the microscope, with rapid sintering of silver-related species and could not be confidently examined. Hence, only different views of the calcined Ag/SBA-15 reacted with molecular iodine are shown on Figure 8A–D. These images can be compared with those of the non-reacted sorbent displayed on Figure 3A,B. The features observed were rather heterogeneous depending on the examined zones. Although rather intact mesopores remained, a part of the ordered pore structure of the SBA-15 support collapsed after exposure to iodine. These damages are caused by the rapid coalescence of AgI entities within the SBA-15 mesopores. Hence, the dark areas in the images shown on Figure 8A–D are partly associated to silver iodide precipitates of different sizes and types. It is clear that confined AgI precipitates in the mesoporosity with size less than 6–7 nm coexist with larger precipitates on the external surface, with a mean size 10–100 nm. In the course of the silver–iodine reaction, AgI crystalline species progressively formed by solid-state diffusion of dissociated iodine atoms from the surface to the bulk of the Ag^0^ nanoparticles confined in the mesopores. This process induces steric constraints, which are responsible for the enlargement and the partial destruction of the mesopores. In absence of confinement in the pores, AgI precipitates further coalesce and rapidly grow to yield larger precipitates, which can be easily detected by XRD and DRS-UV-Vis. Complementary EDX analyses were performed at different locations of the images. Even when the darkest areas were subjected to chemical analysis, the computed atomic I/Ag ratio were found to greatly differ, with values ranging from 0 to almost 1, and often close to 0.2–0.3, which is not expected for pure AgI. According to our experience, it seems that a partial reduction of AgI to metallic silver occurred under the rather reducing conditions of observation: 2 AgI → 2 Ag +I_2_. It is also well known in photography that AgI species are very sensitive to light, this process also leading to silver reduction. Despite all precautions taken to handle the samples in the dark, it cannot be warranted that photo-reduction has not participated in the partial reduction of AgI to metallic silver. Finally, attempts made at high resolution to distinguish between AgI entities and metallic silver from their exposed crystalline fringes also failed because they have approximately the same *d*-spacing.

Switching back to DRS-UV-Vis characterization of the spent adsorbents, the spectral features obtained after adsorption of molecular iodine could also now be compared with those recorded after CH_3_I adsorption (Figure 7B). Rather, similar spectral characteristics are observed for CH_3_I in comparison with I_2_ adsorption (Figure 7A). Nevertheless, it appears that more important differences could now be observed before and after calcination. In a previous part, it was stated that the CH_3_I adsorption capacities depend mostly on the oxidation state of silver and its dispersion on the surface of the supports, the preferred silver form being Ag^+^. For calcined samples, the presence of remaining residual silver nanoparticles was also noticed by XRD in the case of CH_3_I adsorption (Figure 6). From the spectra gathered in Figure 7B, it could be outlined that calcined silver ceria and alumina materials display (i) a less pronounced absorption edge and at lower wavelength in comparison with their non-calcined counterparts, this meaning the formation of smaller AgI precipitates, and (ii) a strong absorption in the visible region after the absorption edge, indicating that silver nanoparticles have only partially reacted with CH_3_I (see also the DR-UV-Vis spectra of non-treated materials on Figure 2 for comparison). Hence, the silver metallic state seems not very favorable to the adsorption of CH_3_I molecules, which have to undergo dissociation; then, the released I atoms have to diffuse from the surface to the bulk to form larger AgI precipitates. Moreover, this solid-state diffusion process may be partially inhibited by the formation of an AgI layer around the metallic core or by the presence of organic by-products. By contrast, the non-calcined adsorbents exhibit a much more pronounced absorption edge and the presence of residual Ag^+^ cations (around 210 nm, Figure 7B).

## 4. Conclusions

In the future, it is important to progress toward a more rational design of silver-based adsorbents for the capture of volatile iodine species. In this study, the CH_3_I and I_2_ adsorption performances of silver impregnated materials (with 15–18 wt % Ag) made from different host supports (faujasite Y zeolite, SBA-15 mesoporous silica, ceria, and alumina) were compared, and some relationships were established with the physicochemical characteristics of the materials. In order to further tune the silver dispersion and oxidation state on each support, the synthesized adsorbents were either only dried at 80 °C under vacuum or subsequently thermally treated at 500 °C under air. 

In-depth characterizations by XRD, DRS-UV-Vis, and TEM allowed us to precisely determine the silver speciation and dispersion on the different supports. Immediately after drying at 80 °C, the adsorbents were found to display a rather good dispersion of Ag species, with a very high proportion of the silver being present in the +1 oxidation state on Y zeolite and SBA-15 silica. By contrast, silver species on ceria and alumina supports were characterized by a lower degree of dispersion, with a higher proportion of small clusters and reduced nanoparticles, Ag^+^ cations being still the major silver species. 

Depending on the support’s nature, the calcination treatment at 500 °C had adverse effects on the silver speciation and dispersion. Thanks to its high cationic exchange capacity, the faujasite Y zeolite displayed a much higher tendency to stabilize silver as Ag^+^ cations and to a lesser extent as charged and/or metallic clusters within its microporous system. The other supports bearing no exchange sites displayed comparatively a much higher proportion of agglomerated silver nanoparticles in reduced or partially reduced state. On ceria and alumina supports with no internal porosity, the calcination treatment induced a rapid coalescence and self-reduction of Ag species, because of the enhancement of their surface mobility. By contrast, the presence of ordered mesopores in SBA-15 partially restricted the tendency of silver for uncontrolled sintering. 

Depending on the nature of the adsorbate, different trends were obtained for the adsorption performances. On the one hand, it was shown that I_2_ adsorption is not really sensitive to the type of silver species, since it proceeded with a rather similar efficiency both on Ag^+^ cations (at the exchange sites) in the porosity and on agglomerated metallic silver. For each calcined or non-calcined sorbent, the formation of stable AgI precipitates was found to be quantitative with a rather complete use of silver (I/Ag ratio about 1). Hence, adsorption capacities for molecular iodine are mostly a function of the total silver content. 

For CH_3_I adsorption, a very different behavior was observed. CH_3_I adsorption capacities were found to depend mainly on the amount of silver available in oxidized (cationic) form, rather than simply the silver content. AgI precipitates also formed rather easily from the reaction of methyl iodide with Ag^+^ cations, but much less effectively with metallic silver. In that respect, post-characterization of the spent sorbents after CH_3_I test revealed that partially unreacted silver nanoparticles co-exist with AgI for silica, ceria and alumina but not on Ag/Y zeolite. Therefore, it seems that solid-state diffusion processes leading to the progressive conversion of metallic silver to silver iodide in the presence of CH_3_I are somewhat inhibited by the accumulation of organic by-products or by the difficulty to dissociate the C-I bond.

## Figures and Tables

**Table 1:** Main physiochemical characteristics of the investigated adsorbents.

Adsorbent	%Ag	Silver Speciation
14.4 Ag/Al_2_O_3_	14.4	**Ag**^+^, Ag_n_^δ+^, Ag^°^_m_, traces of Ag^°^
14.4 Ag/Al_2_O_3_-calc	14.4	Ag^°^ (**27 nm**)
15.5 Ag/SBA-15	15.5	**Ag**^+^, Ag_n_^δ+^, Ag^°^_m_, traces of Ag^°^
15.5Ag/SBA-15-calc	15.5	Ag^°^ (**9 nm**)
17 Ag/Y (2.5)	17	**Ag**^+^, Ag_n_^δ+^, Ag^°^_m_, traces of Ag^°^
17Ag/Y (2.5)-calc	17	**Ag**^+^, Ag_n_^δ+^, Ag^°^_m_
18.6 Ag/CeO_2_	18.6	n.d.
18.6Ag/CeO_2_-calc	18.6	Ag^°^ (**106 nm**)

**Table 2:** Adsorption characteristics of the investigated calcined and non-calcined silver supports after I_2_ adsorption test (the major phase is indicated in bold).

Adsorbent	Q_I2_ (mg/g)	I/Ag	Color after I_2_ Exposure	Detected Phases by XRD	D AgI Miersite Phase (nm)
14.4 Ag/Al_2_O_3_	177	1.04	Yellow	**AgI Miersite** AgI iodargyrite	37
14.4 Ag/Al_2_O_3_-calc	196	1.16	Black	**AgI Miersite** AgI iodargyrite	49
15.5 Ag/SBA-15	159	0.86	Yellow	**AgI Miersite** AgI iodargyrite	64
15.5Ag/SBA-15-calc	172	0.94	Yellow	**AgI Miersite** AgI iodargyrite	16
17 Ag/Y (2.5)	205	1.02	Pale yellow	**AgI Miersite**	37
17Ag/Y (2.5)-calc	208	1.03	Pale yellow	**AgI Miersite**	35
18.6 Ag/CeO_2_	224	1.02	Yellow	Small peaks **AgI Miersite** AgI iodargyrite	16
18.6Ag/CeO_2_-calc	253	1.15	Grey	**AgI Miersite** AgI iodargyrite	26

**Table 3:** Adsorption properties toward CH_3_I of the investigated calcined and not calcined silver-impregnated supports (the major AgI phase is indicated in bold, the symbol **** means that the presence of AgI or Ag° phases were not detected).

Adsorbent	Q_CH_3_I_ (mg/g)	I/Ag	Detected Phases by XRD	D AgI Miersite Phase (nm)	D Ag° (nm)
14.4 Ag/Al_2_O_3_	122	0.64	**AgI Miersite** AgI iodargyrite	44	****
14.4 Ag/Al_2_O_3_-calc	38	0.2	Ag° nanoparticles	Small AgI peaks	28
15.5 Ag/SBA-15	143	0.7	**AgI Miersite** AgI iodargyrite	73	****
15.5 Ag/SBA-15-calc	61	0.3	Ag° nanoparticles	Small AgI peaks	30
17 Ag/Y (2.5)	150	0.67	**AgI Miersite**	73	****
17 Ag/Y (2.5)-calc	144	0.64	**AgI Miersite**	58	****
18.6 Ag/CeO_2_	189	0.77	**AgI Miersite** AgI iodargyrite	39	****
18.6 Ag/CeO_2_-calc	22	0.09	Ag° nanoparticles	****	29

## Data Availability

The data presented in this article are available on request from the corresponding author.

## Supplementary Materials

The following are available online at https://www.mdpi.com/article/10.3390/nano11051300/s1, Table S1: Experimental conditions of the ionic exchange reaction for all the prepared silver zeolites materials, Table S2: Silver analysis by Atomic Absorption Spectrometry (AAS), S3: Determination of cystallites mean size by the Debye–Scherrer formula, Figure S1: Some N_2_ adsorption isotherms of the investigated silver loaded sorbents.

## References

[1] Clement B., Cantrel L., Ducros G., Funke F., Herranz L., Rydl A., Weber G., Wren C. (2007). State of the art report on iodine chemistry. OECD Report, NEA/CSNI/R.

[2] Haefner D.R., Tranter T.J. (2007). Methods of Gas Phase Capture of Iodine from Fuel Reprocessing Off-Gas: A Literature Survey. INL/EXT-07-12299.

[3] Cantrel L., Herranz L.E., Guieu S., Albiol T., Collet R., Lind T., Karkela T., Mun C., Jacquemain D., Chebbi M. Overview of ongoing and planned R&D works on delayed releases and FCVS efficiencies. Proceedings of the ICAPP 2015.

[4] Ikemoto T., Magara Y. (2011). Measures against impacts of nuclear disaster on drinking water supply systems in Japan. Water Pract. Technol..

[5] Maeck W.J., Pence D.T., Keller J.H. (1969). A Highly Efficient Inorganic Adsorber for Airborne Iodine Species (Silver Zeolites Development Studies).

[6] Pence D.T., Duce F.A., Maeck W.J., First M.W. (1972). Developments in the Removal of Airborne Iodine Species with Metal Substituted Zeolites. Proceedings of the 12th AEC Air Cleaning Conference.

[7] Thomas T.R., Staples B.A., Murphy L.P., Nichols J.T. (1977). Airborne Elemental Iodine Loading Capacities of Metal Exchanged Zeolites and a Method for Recycling Silver Zeolites.

[8] Jubin R.T. (1979). A Literature Survey of Methods to Remove Iodine from Off-Gas Streams Using Solid Sorbents.

[9] Herranz L.E., Lind T., Dieschbourg K., Riera E., Morandi S., Rantanen P., Chebbi M., Losch N. State of the Art Report: Technical Bases for Experimentation on Source Term Mitigation Systems. Proceedings of the 10th International Topical Meeting on Nuclear Thermal Hydraulics, Operation and Safety (NUTHOS-10).

[10] Huve J., Ryzhikov A., Nouali H., Lalia V., Augé G., Daou T.J. (2018). Porous sorbents for the capture of radioactive iodine compounds: A review. RSC Adv..

[11] Chebbi M. (2016). Piégeage D’espèces Iodées Volatiles sur des Adsorbants Poreux de Type Zéolithique dans le Contexte d’un Accident Nucléaire Grave. Ph.D. Thesis.

[12] Chebbi M., Azambre B., Cantrel L., Koch A. (2016). A combined DRIFTS and DR-UV–Vis spectroscopic in situ study on the trapping of CH_3_I by silver-exchanged faujasite zeolite. J. Phys. Chem. C.

[13] Chebbi M., Chibani S., Paul J.F., Cantrel L., Badawi M. (2017). Evaluation of volatile iodine trapping in presence of contaminants: A periodic DFT study on cation exchanged-faujasite. Microporous Mesoporous Mater..

[14] Chebbi M., Azambre B., Cantrel L., Huvé M., Albiol T. (2017). Influence of structural, textural and chemical parameters of silver zeolites on the retention of methyl iodide. Microporous Mesoporous Mater..

[15] Azambre B., Chebbi M. (2017). Evaluation of silver zeolites sorbents toward their ability to promote stable CH_3_I storage as AgI Precipitates. ACS Appl. Mater. Interfaces.

[16] Azambre B., Chebbi M., Leroy O., Cantrel L. (2018). Effects of zeolitic parameters and irradiation on the retention properties of silver zeolites exposed to molecular iodine. Ind. Eng. Chem. Res..

[17] Azambre B., Chebbi M., Hijazi A. (2020). Effects of the cation and Si/Al ratio on CH_3_I adsorption by faujasite zeolites. Chem. Eng. J..

[18] Chebbi M., Azambre B., Monsanglant-Louvet C., Marcillaud B., Roynette A., Cantrel L. (2021). Effects of water vapour and temperature on the retention of radiotoxic CH_3_I by silver faujasite zeolites. J. Hazard. Mater..

[19] Jacquemain D., Guentay S., Basu S., Sonnenkalb M., Lebel L., Allelein H.J., Liebana B., Eckardt B., Ammirabile L. (2014). Status Report on Filtered Containment Venting. OECD/NEA/CSNI, Report NEA/ CSNI/R.

[20] Herrmann F.J., Herrmann B., Hoeflich V., Beyer C.H., Furrer J. Removal Efficiency of Silver Impregnated Filter Materials and Performance of Iodine Filters in of the Off-Gases of the Karlsruhe Reprocessing Plant WAK. Proceedings of the 24th DOE/NRC Nuclear Air Cleaning and Treatment Conference.

[21] Fukasawa T., Funabashi K., Kondo Y. Influences of impurities on iodine removal efficiency. Proceedings of the 24th DOE/NRC Nuclear Air Cleaning Conference and Air Treatment.

[22] Wilhelm J.G., Furrer J. Head-end iodine removal from a reprocessing plant with a solid sorbent. Proceedings of the ERDA 14th Air Cleaning Conference, CONF720823.

[23] IAEA Report (1987). Treatment, Conditioning and Disposal of Iodine-129.

[24] Matyáš J., Fryxell G.E., Busche B.J., Wallace K., Fifield L.S., Lin H.-T., Katoh Y., Fox K.M., Belharouak I., Widjaja S., Singh D. (2011). Ceramic Materials for Energy Applications: Ceramic Engineering and Science.

[25] Mnasri N., Charnay C., Ménoval L., Moussaoui Y., Elaloui E., Zajac J. (2014). Silver nanoparticle-containing submicron-in-size mesoporous silica-based systems for iodine entrapment and immobilization from gas phase. Microporous Mesoporous Mater..

[26] Zhao D., Feng J., Huo Q., Melosh N., Fredrickson G.H., Chmelka B.F., Stucky G.D. (1998). Triblock copolymer syntheses of mesoporous silica with periodic 50 to 300 angstrom pores. Science.

[27] Hijazi A., Azambre B., Finqueneisel G., Vibert F., Blin J.L. (2019). High iodine adsorption by polyethyleneimine impregnated nanosilica sorbents. Microporous Mesoporous Mater..

[28] Trovarelli A. (1996). Catalytic Properties of Ceria and CeO_2_-Containing Materials. Catal. Rev..

[29] Flanigen E.M., Van Bekkum H., Flanigen E.M., Jansen J.C. (1991). Introduction to Zeolite Science and Practice, Studies in Surface Science and Catalysis.

[30] Bartolomeu R., Azambre B., Westermann A., Fernandes A., Bertolo R., Issa Hamoud H., Henriques C., Da Costa P. (2014). Investigation of the nature of silver species on different Ag-containing NO_x_ reduction catalysts: On the effect of the support. Appl. Catal. B Environ..

[31] Salles N. (2014). Etude des Différents Polymorphes de L’Alumine et des Phases Transitoires Apparaissant lors des Premiers Stades D’Oxydation de L’Aluminium: Simulation à L’Échelle Atomique par un Modèle à Charges Variables en Liaisons Fortes. Ph.D. Thesis.

[32] Kanipandian N., Kannan S., Ramesh R., Subramanian P., Thirumurugan R. (2014). Characterization, antioxidant and cytotoxicity evaluation of green synthesized silver nanoparticles using Cleistanthus collinus extract as surface modifier. Mater. Res..

[33] Sayah E., Brouri D., Wu Y., Musi A., Da Costa P., Massiani P. (2011). A comparative in situ TEM and UV—Visible spectroscopic study of the thermal evolution of Ag species dispersed on Al_2_O_3_ and NaX zeolite supports. Appl. Catal. A Gen..

[34] Aspromonte S.G., Mizrahi M.D., Schneeberger F.A., Lopez J.M.R., Boix A.V. (2013). Study of the nature and location of Silver in Ag-exchanged mordenite catalysts. Characterization by spectroscopic techniques. J. Phys. Chem. C.

[35] Shibata J., Takada Y., Shichi A., Satokawa S., Satsuma A., Hattori T. (2004). Influence of zeolite support on activity enhancement by addition of hydrogen for SCR of NO by propane over Ag-zeolites. Appl. Catal. B Environ..

[36] Baker M.D., Ozin G.A., Godber J. (1985). Far-infrared studies of silver atoms, silver ions, and silver clusters in zeolites A and Y. J. Phys. Chem..

[37] Funke F., Greger G.-U., Bleier A., Hellmann S., Morell W. (1996). The Reaction between Iodine and Silver under Severe PWR Accident Conditions. An Experimental Parameter Study. PSI-97-02, 28035729 Report.

[38] Andryushechkin B.V., Zhidomirov G.M., Eltsov K.N., Hladchanka Y.V., Korlyukov A.A. (2009). Local structure of the Ag(100) surface reacting with molecular iodine: Experimental and theoretical study. Phys. Rev. B.

[39] Bruffey S.H., Jubin R.T., Jordan J.A. (2016). Capture of elemental and organic iodine from dilute gas streams by silver-exchanged mordenite. Proc. Chem..

[40] Zhou X.-L., Solymosi F., Blass P.M., Cannon K.C., White J.M. (1989). Interactions of methyl halides (Cl, Br, and I) with Ag(111). Surf. Sci..

[41] Chapman K.W., Chupas P.J., Nenoff T.M. (2010). Radioactive iodine capture in silver-containing mordenites through nanoscale silver iodide formation. J. Am. Chem. Soc..

[42] Kodaira T., Ikeda T., Takeo H. (1999). Optical and X-ray diffraction study of AgI clusters incorporated into zeolite LTA. Eur. Phys. J. D.

