# Supplementary material for: Structure–Activity Relationships between the State of Silver on Different Supports and Their I2 and CH3I Adsorption Properties
Source: Nanomaterials (Basel). 2021 May 14;11(5):1300. doi: 10.3390/nano11051300 (PMC8155951; doi:10.3390/nano11051300)

Article

Structure-activity relationships between the state of silver on different supports and their I_2 and CH_3I adsorption properties

Bruno Azambre [1,*], Mouheb Chebbi [2] and Nagham Ibrahim [1]

[1] Université de Lorraine, Laboratoire de Chimie et Physique-Approche Multi-Echelle des Milieux Complexes (LCP-A2MC- EA n°4362), Institut Jean-Barriol FR2843 CNRS, ICPM, 1, Bd Arago, F-57500 Saint-Avold, France; bruno.azambre@univ-lorraine.fr
[2] Institut de Radioprotection et de Sûreté Nucléaire (IRSN), PSN-RES, Saclay, 91192 Gif-sur-Yvette, France; mouheb.chebbi@irsn.fr
* Correspondence: bruno.azambre@univ-lorraine.fr;

Supporting information

Table S1: Experimental conditions of the ionic exchange reaction for all the prepared silver zeolites materials

Support	V porous (mL/g)	$AgNO_3$ mass (g)
**SBA-15**	-	-
**NH_4/Y (2.5)**	0.8	1.96
**CeO_2**	0.3	5.25
**Al_2O_3**	0.7	2.25

Table S2: Silver analysis by Atomic Absorption Spectrometry (AAS)

First, a solubilization step was conducted using a concentrated nitric acid solution. A fixed mass of the sorbent was kept in contact with the HNO_3 solution (50 mL, 1.6 mol.L^{-1}) for a duration of 24 hours. Then, 1 mL of solution was recovered using a micro-filtre, and diluted with a dilution factor of 100. Finally, the obtained solution was analysed by AAS, using an air acetylene flame (T_{max} about 2600 K). The related analytical parameters are summarized in the following table :

Element	λ (nm)	Analysis mode	Lamp and parameters	Calibration equation
**Ag**	338.3	Absorption	AgCuCr *(13170407)*, 75% and 10	$y = 0.07765 \times x - 0.0077$

S3: Determination of cystallites mean size by the Debye-Scherrer formula

In the used formula, the line broadening related to the instrument was taken into account :

$$D = \frac{K \times \lambda}{\cos\theta \times \sqrt{L^2 - L^2{}_{inst}}}$$

Where :

- D : Cristallites mean size (nm) ;
- K : the dimensionless shape factor (fixed to a value of 0.9 in the present study) ;
- λ : the X-ray Wavelength : 0.15418 nm ;
- θ : the Bragg angle (rad) ;
- L : the full width at half maximum (FWHM) of the pic related to 2θ (rad) ;
- L_{inst} : the instrumental line boradenig (0.07° = for the used device).

Figure S1: Some N_2 adsorption isotherms of the investigated silver loaded sorbents

(A) SBA-15 materials

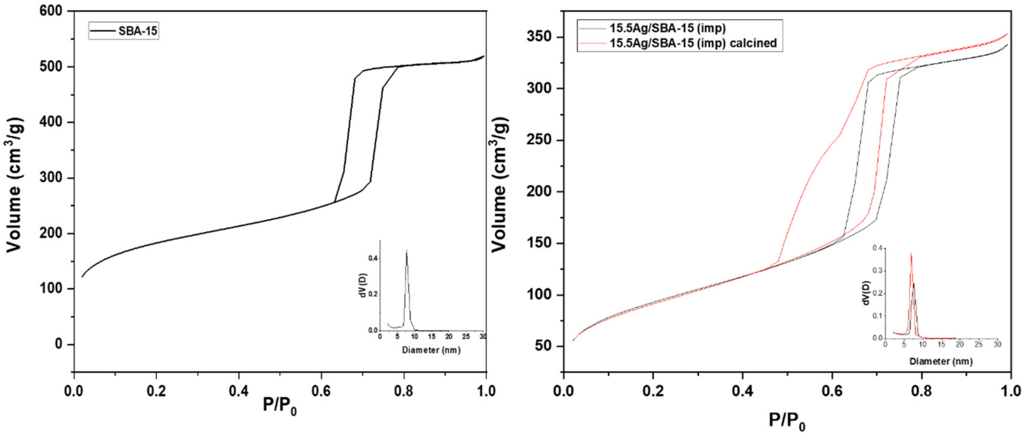

(B) Ag-Alumina, Ag-Ceria and Ag-Y

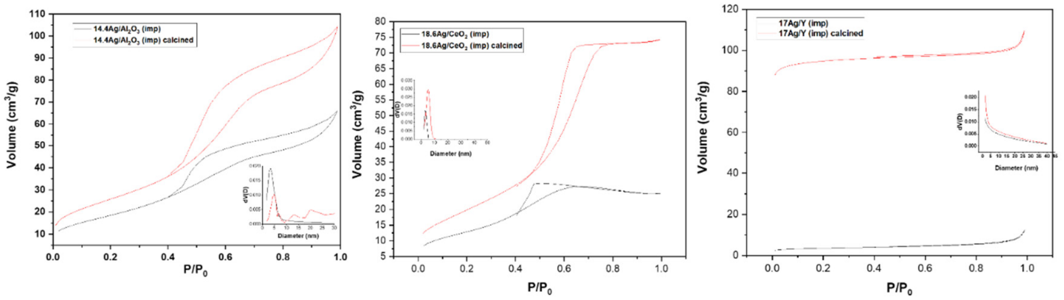

Supplement: Supplementary file 1 [file nanomaterials-11-01300-s001.zip › nanomaterials-1207474-supplementary.pdf]
